# Correlation between the mechanism of arteriopathy in IgA nephropathy and blood stasis syndrome: A cohort study

**DOI:** 10.1515/med-2024-1042

**Published:** 2024-12-09

**Authors:** Ruiqi Wang, Yun Tian

**Affiliations:** Department of Traditional Chinese Medicine, Shaanxi Provincial Hospital of Traditional Chinese Medicine, Xi’an, 710003, China; Department of Nephrology, Shaanxi Provincial Hospital of Traditional Chinese Medicine, No. 4, Xihuamen, Xi’an, Shaanxi, 710003, China

**Keywords:** IgAN, arteriopathy, correlation, blood stasis syndrome

## Abstract

To investigate the correlation between blood stasis syndrome and arteriopathy in immunoglobulin A nephropathy (IgAN). Wall thickness/outer vessel diameter, intimal thickness/outer vessel diameter, and medial thickness/outer vessel diameter were measured using ImageJ software. Vascular endothelial-derived growth factor (VEGF), matrix metalloproteinase-9 (MMP-9), proliferating cell nuclear antigen (PCNA), extracellular signal-regulated kinase (ERK) 1/2, and nuclear factor kappa B (NF-κB) were detected by immunohistochemical staining. Twenty-four-hour urine protein quantification, serum creatinine, urea nitrogen, and uric acid were collected. Blood stasis syndrome and vessel scores were calculated based on Katafuchi’s grade. Intimal thickness/outer vessel diameter (0.2725 ± 0.0932 μm), medial thickness/outer vessel diameter (0.2747 ± 0.1139 μm), and wall thickness/outer vessel diameter (0.6136 ± 0.1120 μm) were the largest in IgAN with arteriopathy group. VEGF (0.35 ± 0.90), MMP-9 (0.38 ± 0.12), PCNA (0.43 ± 0.12), ERK1/2 (0.31 ± 0.11), and NF-κB (0.37 ± 0.14) were the highest in IgAN with arteriopathy group. Intimal thickening of IgAN was moderately positively correlated with VEGF, MMP-9, PCNA, ERK1/2, and NF-κB (0.5 < *r* < 0.8). Medial thickening of IgAN was moderately positively correlated with PCNA and NF-κB (0.5 < *r* < 0.8). Wall thickening of IgAN was lowly positively correlated with VEGF and MMP-9 (0.3 < *r* < 0.5). Blood stasis syndrome score was associated with vessel score in IgAN with arteriopathy (*P* < 0.05). Blood stasis syndrome score can assess the degree of pathological changes.

## 1 Introduction

IgA nephropathy (IgAN) is the most common primary glomerular disease worldwide, which occurs frequently in Asia [1]. The pathogenesis of IgAN is related to heredity, immunity, inflammation, and alternative factors. Previous studies of the pathogenesis of IgAN mostly focus on glomerular, renal tubular, and renal interstitial lesions, and the latest Oxford classification of IgAN also includes these parameters. Vascular disease is also one of the important factors to accelerate the progression of IgAN [2], and the proportion of arteriopathy is gradually increasing in clinical practice. Nevertheless, its influence on clinical prognosis remains controversial. Therefore, the latest Oxford classification has not included arterioles-related parameters. Previous diagnostic criteria of renal arteriolar lesions comprise renal arteriolar sclerosis (arteriolar hyalinization, intimal thickening) and renal arteriolar microvascular lesions (arteriolar and or minimal endothelial damage). Clinical studies have also found that the control of blood pressure in patients with IgAN complicated with arteriopathy is poor, with a rapid increase of proteinuria and renal function injury [3]. Therefore, pathological changes in renal arterioles in IgAN can be used as an independent risk factor for the end-point event of IgAN.

At present, the mechanism underlying intrarenal arteriolar lesions in IgAN remains elusive, and it is widely believed that arteriolar lesions in IgAN are closely related to malignant hypertension. However, a large sample-size follow-up conducted by the Peking University Health Science Center suggests that the probability of microangiopathy in non-hypertensive IgAN patients is roughly 8.8% [4]. Liu et al. have found that the pathological mechanism of IgAN does not entirely depend on hypertension [5]. According to the characteristics of IgAN arteriopathy combined with literature review, Chinese scholars from Shaanxi Provincial Hospital of Chinese Medicine put forward a prospective assumption that whether the pathogenesis of IgAN arteriopathy is correlated with the activation of renin–angiotensin–aldosterone system, especially the activation of extracellular signal-regulated kinase/nuclear factor kappa B (ERK/NF-κB) signaling pathway caused by aldosterone increase *in vivo*, leading to the increase of matrix metalloproteinase-9 (MMP-9), proliferating cell nuclear antigen (PCNA), and vascular endothelial-derived growth factor (VEGF) levels.

Blood stasis syndrome refers to a series of clinical syndromes manifested with blood stasis. From the pathological perspective, the thickening of arteriolar wall, stenosis, and vitreous degeneration of IgAN arteriopathy will all cause poor blood flow in arteriolar lumen and stagnation, which corresponds to the intravascular stasis syndrome of modern Chinese medicine. Patients with arteriopathy in IgAN often have a dark complexion, dark purple tongue, ecchymosis, and persistent low back pain, corresponding to extravascular stasis syndrome in modern Chinese medicine [6]. Previous large-sample follow-up studies have suggested that blood stasis syndrome is the most common syndrome in IgAN, accounting for 28.9% [7]. Blood stasis plays an important role in the deterioration and progression of IgAN arteriopathy. Hence, it is necessary to unravel the correlation between IgAN arteriopathy and blood stasis syndrome.

In the present study, the intimal, medial, and vascular wall thickness of IgAN arterioles were measured. The correlation between intimal, medial, and vascular wall thickening and PCNA, VEGF, and MMP-9 cytokines was assessed. The relationship between ERK/NF-κB signaling pathway and multiple cytokines was determined, thereby elucidating the mechanism of pathological changes in IgAN arterioles. Combined with blood stasis syndrome, the blood stasis syndrome scores were calculated and compared, the correlation between IgAN blood stasis syndrome score and vessel score was analyzed, and the correlation between blood stasis syndrome and IgAN arteriopathy was discussed, aiming to provide a basis for potential clinical treatment.

## 2 Materials and methods

### 2.1 Patients

From September 2019 to March 2022, 60 patients diagnosed with IgAN by biopsy with normal blood pressure and 30 patients without IgAN with mild mesangial hyperplasia/minimal lesions in Shaanxi Provincial Hospital of Chinese Medicine were included in this study. According to the results of the pathological biopsy, all patients were divided into the IgAN with arteriopathy group, IgAN without arteriopathy group, and non-IgAN group, respectively.

### 2.2 Inclusion criteria

Sixty patients were diagnosed with primary IgAN by renal biopsy; 30 patients were diagnosed with non-IgAN by renal biopsy presenting with mild mesangial hyperplasia/minimal lesions; age ≥18 years old; the number of renal biopsy puncture was ≥10; and the sagittal section of arterioles was ≥3.

### 2.3 Exclusion criteria

Those with secondary nephropathy, such as systemic lupus nephritis, small vasculitis renal damage, henoch-schonlein purpura nephritis, hepatitis B virus-related nephritis, hypertensive renal damage, diabetic nephropathy, obesity-related nephropathy, and those with a medical history of diabetes mellitus, smoking, and hypertension were excluded.

### 2.4 Laboratory index

Twenty-four-hour urine protein quantification (24hU-TP): 24-h urine samples were collected once before renal puncture and detected by pyrophenol red method with automatic biochemical analyzer, and the average value was calculated. A portion of 3 ml of blood samples was collected under fasting in the morning for the detection of serum creatinine (Scr), urea nitrogen (BUN), and uric acid (UA), injected into a tube, centrifuged at 4°C and 3,000 rpm for 10 min, and the supernatant was collected for subsequent testing. All the above laboratory indexes were performed by the Clinical Laboratory of Shaanxi Provincial Hospital of Chinese Medicine.

### 2.5 Pathological data of renal biopsy

Renal biopsy tissues were examined by routine immunofluorescence, light microscopy, and electron microscopy and subsequently subject to HE, PASM, PAS, and Masson staining, respectively. Pathological scoring was carried out by an experienced pathologist according to scoring criteria of Oxford classification of IgAN patients updated in 2017.

### 2.6 Measurement of renal arteriole thickness and Katafuchi’s grading of vessel score

PASM staining sections were observed under an electron microscope (×200), and the photos in the middle area of arterioles were taken. The wall thickness/vascular diameter, intimal thickness/outer vascular diameter, medial thickness/outer vascular diameter, wall, intimal, and medial thickness were measured three times, and the average value was calculated. The wall thickening was evaluated according to Katafuchi’s grading. Under the cross section on the same plane, the wall thickness/outer vascular diameter should exceed 0.5. Inner vessel diameter/outer vessel diameter ≤0.5 is defined as vessel wall thickening. According to the percentage of diseased blood vessels, vascular wall thickening of 0% represents 0 points, <10% as 1 point, 10–25% as 2 points, and >25% as 3 points, respectively. The parameter of hyalinization percentage is the same as that of vascular wall thickening. The total score ranges from 0 to 6 points.

### 2.7 Immunohistochemical staining

The expression levels of VEGF, MMP-9, PCNA, ERK1/2, and NF-κB in renal arterioles were detected by two-step immunohistochemical staining using the Envision System. Primary antibodies consisted of VEGF antibody (rabbit anti-human), PCNA antibody (rabbit anti-human), MMP-9 antibody (mouse anti-human), ERK1/2 antibody (rabbit anti-rat), and NF-κB antibody (rabbit anti-rat). After each antibody was added, it was diluted with phosphate-buffered solution (PBS). The dilution ratio of VEGF, MMP-9, and NF-κB antibody was 1:200, and the dilution ratio of PCNA and ERK1/2 antibody was 1:300. Secondary antibody was goat anti-rabbit or anti-mouse antibody, and the marker was horseradish peroxidase (HRP). First, the tissues were dehydrated with gradient alcohol, transparent, soaked in wax, embedded, and sliced. Paraffin sections were dewaxed, antigen was repaired, endogenous peroxidase was blocked, serum was sealed, primary antibody was added dropwise, and incubated overnight (15 h) in a wet box at 4°C. A secondary antibody was added, the slices were rinsed with PBS for 3 min each time, labeled with HRP, incubated at 37°C for 30 min, newly prepared diaminoben-zidine chromogenic agent (brownish yellow) was supplemented, and the nuclei were stained with hematoxylin. A positive signal was seen in brown color and then counterstained, dehydrated, sealed, and photographed under a microscope. All arterioles were photographed under ×200 times, and the gray values of the brown areas of renal tissues and the arterioles were measured by ImageJ software. The positive expression area was stained brown in color. The average optical density (AOD) of arteriole area was calculated by ImageJ software, and AOD refers to the ratio of integrated optical density to positive area (IOD/area).

### 2.8 Blood stasis syndrome score

According to Katafuchi’s grading of IgAN in combination with the blood stasis syndrome score [8], a score of below 15 is classified as non-blood stasis syndrome, 16–30 as mild blood stasis syndrome, 31–50 as moderate blood stasis syndrome, and >50 as severe blood stasis syndrome, respectively. The score was calculated by two physicians with the title of attending physician or above.

### 2.9 Katafuchi’s grading of IgA nephropathy

The glomerular score is 12 points, which consists of 4 points of glomerularhypercellularity (mesangial and endocapillary), 4 points of segmental lesions, and 4 points of global glomerular sclerosis, respectively [9]. The total tubulo-interstitial score is 9 points, which comprises 3 points of interstitial inflammatory cell infiltration, 3 points of interstitial fibrosis, and 3 points of tubular atrophy, respectively. The total vessel score is 6, including 3 points of vascular wall thickening and 3 points of hyalinization.

### 2.10 Lee’s grading system for IgAN

Lee’s grading system for IgAN is defined as follows [10]: grade I, normal or focal mesangial cell proliferation; grade II, diffuse mesangial cell proliferation, or <25% of glomeruli with crescent (Cr)/segmental sclerosis (SS)/global sclerosis (GS); grade III, 25–49% of glomeruli with Cr/SS/GS; grade IV, 50–75% of glomeruli with Cr/SS/GS; and grade V, >75% of glomeruli with Cr/SS/GS.

### 2.11 Statistical analysis

SPSS23.0 software was used for all statistical analyses (SPSS Inc., Chicago, IL). The data conform to normal distribution were expressed by the mean ± standard deviation (SD). Comparison among three groups was performed by one-way analysis of variance for pairwise comparison. The least significant difference test was used for homogeneous variance, and a nonparametric test was employed for heterogeneous variance. The Pearson correlation test was used for normal distribution data and the Spearman correlation test for non-normal distribution data. A *P* value of less than 0.05 was considered as statistically significant.


**Informed consent:** Participants have provided their written informed consent to participate in this study.
**Ethical approval:** This study was approved by The Ethics Committee of Shaanxi Provincial Hospital of Traditional Chinese Medicine.

## 3 Results

### 3.1 Baseline data

**Figure 1:**
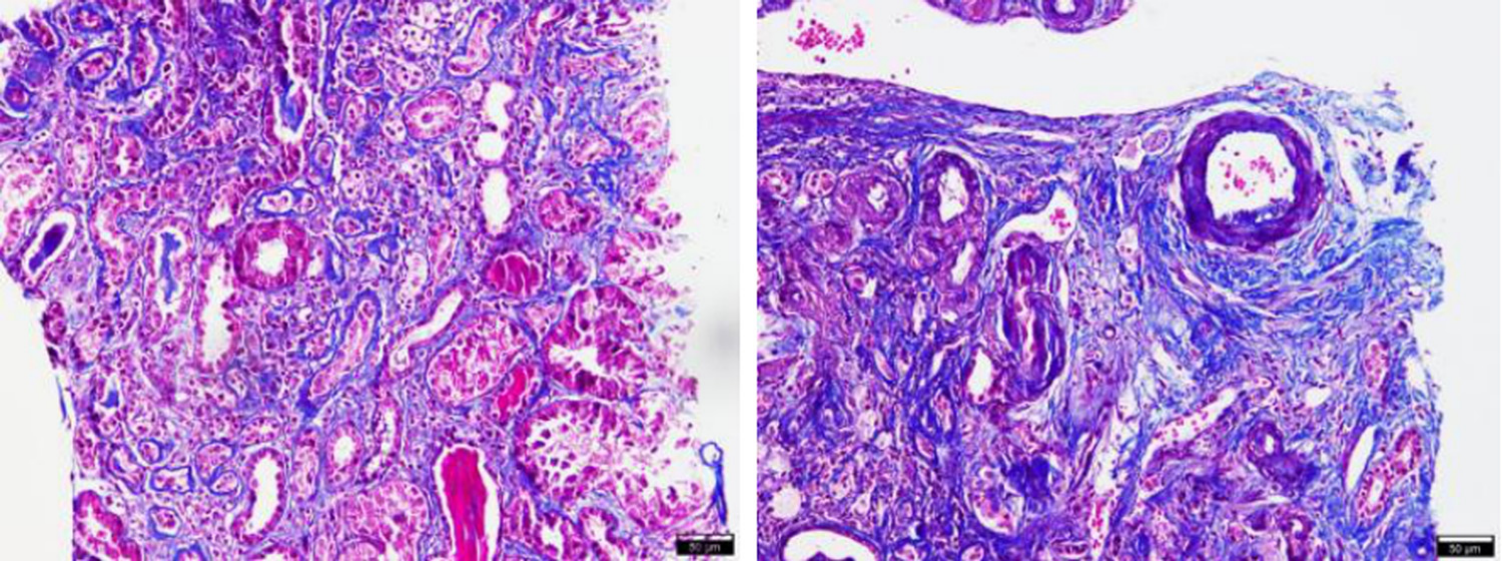
Masson staining of IgAN with arteriopathy.

**Figure 2:**
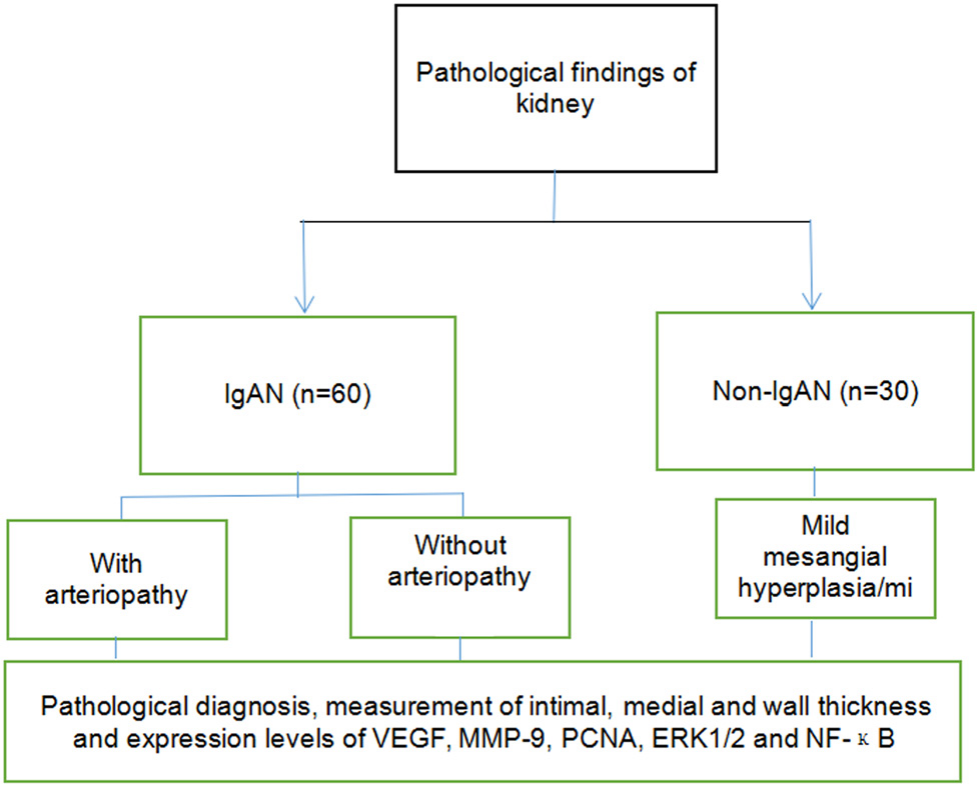
Flow chart of study procedures.

According to the inclusion and exclusion criteria, 60 patients with primary IgAN with normal blood pressure admitted to Shaanxi Provincial Hospital of Chinese Medicine from September 2019 to March 2022 were finally included in this study. Among them, 30 cases of IgAN with arteriopathy under a microscope were assigned to group A (Figure 1), 30 cases without arteriopathy were allocated to group B, and 30 cases without IgAN were included in group C. The study procedures are shown in Figure 2.

### 3.2 Comparison of laboratory parameters

Compared with group B, the levels of 24hU-TP, serum creatinine, and urea nitrogen in group A were significantly higher (all *P* < 0.05), while no statistical significance was observed in the uric acid level between the two groups (*P* > 0.05). Compared with group C, the levels of 24hU-TP, serum creatinine, and urea nitrogen in group A were significantly higher (all *P* < 0.05), but there was no statistical significance in the uric acid level (*P* > 0.05). No significant differences were observed in 24hU-TP, serum creatinine, urea nitrogen, and uric acid levels between groups B and C (all *P* > 0.05), as shown in Table 1.

**Table 1:** Comparison of laboratory parameters among three groups (mean ± SD)

Parameter	Group A	Group B	Group C
24hU-TP (mg/24 h)	1590.5 ± 1459.62*^#^	603.28 ± 318.70	770.56 ± 437.22
Serum creatinine (μmol/L)	96.95 ± 51.71*^#^	70.31 ± 15.16	64.53 ± 17.97
Blood urea nitrogen (mmol/L)	6.35 ± 2.84*^#^	4.79 ± 1.08	4.66 ± 1.32
Uric acid (μmol/L)	369.00 ± 92.01	345.70 ± 77.05	337.42 ± 105.16

Note: *P < 0.05 indicates a significant difference compared with group B; ^#^ *P* < 0.05 indicates a significant difference compared with group C.

### 3.3 Comparison of arteriolar thickness

Compared with group B, the intimal thickness/outer vascular diameter, medial thickness/outer vascular diameter, and wall thickness/outer vascular diameter were increased significantly in group A (all *P* < 0.05). Compared with group C, the intimal thickness/outer vascular diameter, medial thickness/outer vascular diameter, and wall thickness/outer vascular diameter were significantly increased in group A (all *P* < 0.05). Compared with group C, the intimal thickness/outer vascular diameter, medial thickness/outer vascular diameter, and wall thickness/outer vascular diameter did not significantly differ (all *P* > 0.05), as shown in Table 2.

**Table 2:** Comparison of arteriolar thickness (μm) among three groups (mean ± s)

Parameter	Group A	Group B	Group C
Intimal thickness/outer vascular diameter	0.2725 ± 0.0932*^#^	0.1556 ± 0.0604	0.1476 ± 0.0570
Medial thickness/outer vascular diameter	0.2747 ± 0.1139*^#^	0.1684 ± 0.0528	0.1763 ± 0.0879
Wall thickness/outer vascular diameter	0.6136 ± 0.1120*^#^	0.2938 ± 0.0880	0.2634 ± 0.0636

Note: *P < 0.05 indicates a significant difference compared with group B; ^#^ *P* < 0.05 indicates a significant difference compared with group C.

### 3.4 Comparison of expression levels of VEGF, MMP-9, PCNA, ERK1/2, and NF-κB

**Figure 3:**
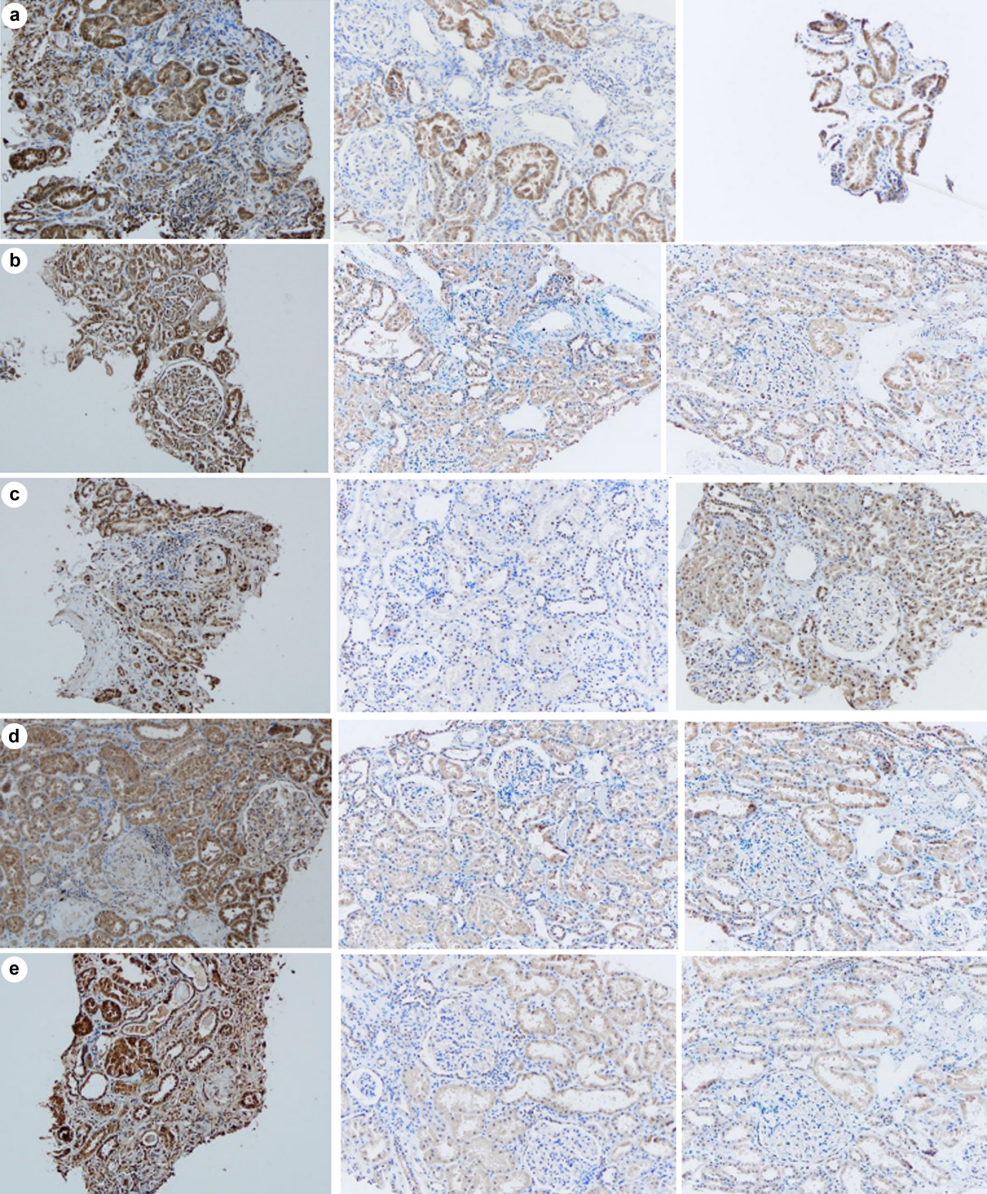
Expression levels of ERK1/2 (a), NF-κB (b), PCNA (c), VEGF (d), and MMP-9 (e) in three groups (×200).

Compared with group B, the expression levels of ERK1/2, MMP-9, NF-κB, PCNA, and VEGF were significantly higher in group A (all *P* < 0.01). Compared with group C, the expression levels of ERK1/2, MMP-9, NF-κB, PCNA, and VEGF were significantly up-regulated in group A (all *P* < 0.01). No significant differences were observed in the expression levels of ERK1/2, MMP-9, NF-κB, PCNA and VEGF between groups B and C (all *P* > 0.05), as illustrated in Table 3 and Figure 3.

**Table 3:** Comparison of expression levels of VEGF, MMP-9, PCNA, ERK1/2 and NF-κB among three groups (mean ± SD)

Group	VEGF	MMP-9	PCNA	ERK1/2	NF-κB
Group A	0.35 ± 0.90*^#^	0.38 ± 0.12*^#^	0.43 ± 0.12*^#^	0.31 ± 0.11*^#^	0.37 ± 0.14*^#^
Group B	0.15 ± 0.02	0.15 ± 0.02	0.16 ± 0.02	0.13 ± 0.02	0.16 ± 0.02
Group C	0.15 ± 0.03	0.13 ± 0.01	0.14 ± 0.02	0.13 ± 0.02	0.15 ± 0.02

Note: *P < 0.05 indicates a significant difference compared with group B; ^#^ *P* < 0.05 indicates a significant difference compared with group C.

### 3.5 Correlation between arteriolar thickness and expression levels of VEGF, MMP-9, PCNA, ERK1/2, and NF-κB

Intimal thickening of IgAN was positively correlated with the expression levels of VEGF, MMP-9, PCNA, ERK1/2, and NF-κB (0.5 < *r* < 0.8). Medial thickening of IgAN was positively correlated with the expression levels of PCNA and NF-κB (0.5 < *r* < 0.8), whereas negatively correlated with those of VEGF, MMP-9, and ERK1/2 (0.3 < *r* < 0.5). Wall thickening of IgAN was positively correlated with those of VEGF and MMP-9 (0.3 < *r* < 0.5), as shown in Table 4.

**Table 4:** Correlation between arteriolar thickness and expression levels of VEGF, MMP-9, PCNA, ERK1/2, and NF-κB (mean ± SD)

Vascular lesions	Expression level	Correlation coefficient	*P* value
Intimal thickness/outer vascular diameter	VEGF	0.559	0.001
	MMP-9	0.537	0.002
	PCNA	0.541	0.002
	ERK1/2	0.634	<0.01
	NF-κB	0.658	<0.01
Medial thickness/outer vascular diameter	VEGF	0.462	0.01
	MMP-9	0.328	0.077
	PCNA	0.531	0.003
	ERK1/2	0.462	0.01
	NF-κB	0.59	0.001
Wall thickness/outer vascular diameter	VEGF	0.389	0.034
	MMP-9	0.415	0.023
	PCNA	0.258	0.168
	ERK1/2	0.355	0.054
	NF-κB	0.303	0.104

### 3.6 Correlation analysis of ERK and NF-κB pathway with VEGF, MMP-9, and PCNA

The expression level of ERK1/2 was moderately and positively correlated with those of VEGF and MMP-9 (0.5 < *r* < 0.8), whereas lowly and negatively correlated with the expression level of PCNA (0.3 < *r* < 0.5). The expression level of NF-κB was moderately and positively correlated with the expression levels of VEGF, MMP-9 and PCNA (0.5 < *r* < 0.8), as illustrated in Table 5.

**Table 5:** Correlation analysis of ERK and NF-κB pathway with VEGF, MMP-9, and PCNA

Signaling pathway	Expression level	Correlation coefficient	*P* value
ERK1/2	VEGF	0.596	0.001
	MMP-9	0.793	<0.01
	PCNA	0.426	0.019
NF-κB	VEGF	0.595	0.001
	MMP-9	0.643	<0.01
	PCNA	0.753	<0.01

### 3.7 Correlation analysis of blood stasis syndrome and vascular scores with VEGF, MMP-9, and PCNA

The score of blood stasis syndrome was negatively correlated with PCNA rather than MMP-9, PCNA, VEGF, ERK1/2, and NF-κB. There was no significant correlation between vascular score and MMP-9, PCNA, VEGF, ERK1/2, and NF-κ B expression levels. Considering that cytokines might be more closely correlated with the inflammatory response scores, the micro-inflammatory response score will be calculated in subsequent experiments, as shown in Table 6.

**Table 6:** Correlation analysis of blood stasis syndrome and vascular scores with VEGF, MMP-9, and PCNA

	Expression level	Correlation coefficient	*P* value
Blood stasis syndrome score	MMP-9	0.026	0.891
	PCNA	−0.325	0.08
	VEGF	−0.065	0.732
	ERK1/2	0.19	0.314
	NF-κB	−0.068	0.718
Vascular score	MMP-9	−0.054	0.776
	PCNA	−0.358	0.052
	VEGF	0.027	0.886
	ERK1/2	0.206	0.274
	NF-κB	−0.143	0.45

### 3.8 Comparison of the incidence of blood stasis syndrome among three groups

Among 60 patients with IgAN, 54 cases (90%) were complicated with blood stasis syndrome, including 21 mild cases (35%), 23 moderate cases (38.3%), and 10 severe cases (16.6%). Among 30 non-IgAN patients, 22 cases were complicated blood stasis syndrome (73%), including 13 mild cases (59%), 7 moderate cases (32%), and 2 severe cases (9%), as shown in Table 7.

**Table 7:** Comparison of the incidence of blood stasis syndrome among three groups (*n* = 90)

Group	No	Mild (16–30)	Moderate (31–50)	Severe (51–70)	Total
A	2	5	15	8	30
B	4	16	8	2	30
C	8	13	7	2	30
Total	14	34	30	12	90

### 3.9 Relationship between Lee’s grading system and the number of cases of arteriolar lesions and non-arteriolar lesions in IgAN

Among 60 patients diagnosed with IgAN, 28 cases (46.7%) were classified as grade II, 28 cases of grade III (46.7%), 3 cases of grade IV (5%), and 1 case of grade V, respectively. Lee’s grading system was adopted, as illustrated in Table 8.

**Table 8:** Comparison of Lee’s grading in IgAN patients between two groups (*n* = 60)

Lee’s grading system	Group A	Group B	Total
Grade I	0	0	0
Grade II	6	22	28
Grade III	20	8	28
Grade IV	3	0	3
Grade V	1	0	1
Total	30	30	60

### 3.10 Comparison of blood stasis syndrome scores among three groups

The blood stasis syndrome scores were normally distributed among the three groups. In groups A, B, and C, the average age of patients was (37.00 ± 12.52), (33.47 ± 12.04), and (36.67 ± 10.04) years, and no significant differences were noted among the three groups (all *P* > 0.05). In group A, the score of blood stasis syndrome was calculated as 40.33 ± 15.58, which was significantly higher compared with 25.07 ± 13.79 and 25.53 ± 9.37 in groups B and C (both *P* < 0.05).

## 4 Discussion

With the increasing prevalence of patients with IgAN, the proportion of pathological diagnosis of IgAN arteriolar lesions has also been elevated year by year. Arteriopathy is becoming more and more common in clinical practice. However, the mechanism of IgAN arteriolar lesions is still unclear, and clinicians are still controversial about its mechanism research. At present, Chinese scholars have put forward the following viewpoints on the mechanism of IgAN arteriolar lesions: first, in the process of glomerular injury, mesangial cells proliferate, basement membrane permeability changes, and podocytes are damaged, forming focal segmental glomerular sclerosis or spherical sclerosis, leading to retrobulbar blood supply disorder [11]. Second, infiltration of inflammatory cells in renal interstitial promotes the release of inflammatory mediators and cytokines, which eventually leads to the destruction of blood vessel walls [12]. Third, the deposition of immune complexes, such as IgA on renal arterioles and arterioles, stimulates the release of inflammatory mediators and cytokines, promotes the inflammatory reaction, causes the proliferation of smooth muscle cells, damages vascular endothelial cells, and induces immune damage to blood vessels [13]. Fourth, hypertension, renin–angiotensin, and angiopathy mutually promote each other [14].

Researchers [15] found that in IgAN patients with vascular diseases, after inflammatory factors are activated, endothelial cells, vascular smooth muscle cells (VSMCs), and extracellular matrix are over-synthesized, which can lead to the thickening of the arterial wall. Aldosterone plays a role in vascular oxidative stress. Aldosterone not only directly activates p38 and ERK(1/2) signaling pathways, but also activates NF-κB signaling pathway. The activation of the ERK signaling pathway [16] accelerates the proliferation of VSMCs, and the ERK signaling pathway plays an important role during this process. NF-κB signaling pathway [17] can participate in the pathological process of vascular injury by regulating adhesion, cells, chemokines, angiotensin, and other genes related to proliferation and immune response. At the same time, it can regulate matrix metalloproteinases and enhance VEGF gene transcription. In the process of vascular disease, IgAl molecules with abnormal glycosylation participate in the activation of complement, which leads to abnormal coagulation function, and the formation of “onion-like degeneration” is based on the repeated occurrence of this process and the repair of blood vessels after injury [18]. In fact, renal arteriolar sclerosis, especially hyalinization disease, is a characteristic pathological change in hypertensive patients, but in this study, we found that non-hypertensive patients with IgAN will also be complicated by arteriolar lesions, further confirming that hypertension is not the only factor that causes renal arteriolar lesions in patients with IgAN, but also the incidence of renal arteriolar lesions is high in these populations with normal blood pressure, which may be severely affected by renal function and renal pathological damage. After NF-κB is continuously activated, the nuclear localization signal is exposed, which combines with some specific sequence structures on the nuclear DNA, so that related factors, such as MMP-9 and VEGF, are transcribed. Among them, human MMP-9 [19] is important in regulating extracellular matrix (ECM), which can promote VSMC migration and intimal hyperplasia. VEGF [20] is expressed in vascular-rich tissues of multiple organs and plays an important role in the growth and proliferation of capillary endothelial cells. PCNA [21] can protect the structure and function of blood vessels, and it also plays an important role in initiating cell proliferation.

The pathological mechanism of IgAN arterioles may be related to the activation of ERK1/2 and NF-κB signaling pathways, which promotes the expression of VEGF, MMP-9, and PCNA, causes intima thickening, media swelling, and wall thickening, and leads to onion-like degeneration after repeated vascular injury and repair, which in turn leads to the aggravation of renal vascular ischemia and the rapid progress of renal function. The integral of blood stasis syndrome and vascular score of IgAN arteriopathy are higher than those of the non-arteriopathy group and the non-IgAN group, suggesting that physicians can employ appropriate methods of promoting blood circulation and removing blood stasis in the treatment of IgAN arteriopathy in clinical practice, which may delay the progress of renal dysfunction. These markers may be applied in the diagnosis and treatment of patients with IgAN arteriopathy.

## 5 Conclusions

Taken together, the mechanism of IgAN arteriopathy may be the activation of ERK1/2 and NF-κB signaling pathways, which can up-regulate the expression levels of VEGF, MMP-9, and PCNA, and subsequently lead to the intimal, medial, and vessel wall thickening, increase the proportion of glomerular ischemic sclerosis, and lead to the decrease of renal blood supply, thereby accelerating the progress of intrarenal arteriopathy.

## 6 Summary at a glance

Intimal, medial, and wall thickening of IgAN arteriopathy are associated with VEGF, MMP-9, PCNA, and ERK1/2. In addition, the score of blood stasis syndrome was negatively correlated with PCNA rather than MMP-9, PCNA, VEGF, ERK1/2, and NF-κB.
